# Health Professionals’ and Health Professional Trainees’ Views on Addictive Eating Behaviours: A Cross-Sectional Survey

**DOI:** 10.3390/nu12092860

**Published:** 2020-09-18

**Authors:** Tracy Burrows, Antonio Verdejo-Garcia, Adrian Carter, Robyn M. Brown, Zane B. Andrews, Chris V. Dayas, Charlotte A. Hardman, Natalie Loxton, Priya Sumithran, Megan Whatnall

**Affiliations:** 1 Priority Research Centre for Physical Activity and Nutrition, University of Newcastle, Callaghan, NSW 2308, Australia; megan.whatnall@newcastle.edu.au; 2 School of Health Sciences, Faculty of Health and Medicine, University of Newcastle, Callaghan, NSW 2308, Australia; 3 Turner Institute for Brain and Mental Health, Monash University, Clayton, VIC 3800, Australia; antonio.verdejo@monash.edu (A.V.-G.); adrian.carter@monash.edu (A.C.); 4 Florey Institute of Neuroscience and Mental Health, University of Melbourne, Parkville, VIC 3052, Australia; robyn.brown@florey.edu.au (R.M.B.); zane.andrews@monash.edu (Z.B.A.); 5 Monash Biomedicine Discovery Institute and Department of Physiology, Monash University, Clayton, VIC 3800, Australia; 6 School of Biomedical Sciences & Pharmacy, Faculty of Health and Medicine, University of Newcastle, Callaghan, NSW 2308, Australia; christopher.dayas@newcastle.edu.au; 7 Hunter Medical Research Institute (HMRI), New Lambton Heights, NSW 2305, Australia; 8 Department of Psychology, Institute of Population Health, University of Liverpool, Liverpool L69 7ZA, UK; cah@liverpool.ac.uk; 9 School of Applied Psychology, Griffith University, Brisbane, QLD 4122, Australia; n.loxton@griffith.edu.au; 10 Centre for Youth Substance Abuse Research, University of Queensland, Brisbane, QLD 4072, Australia; 11 Department of Medicine (Austin), University of Melbourne, Heidelberg, VIC 3084, Australia; priyas@unimelb.edu.au; 12 Department of Endocrinology, Austin Health, Heidelberg Heights, VIC 3081, Australia

**Keywords:** addictive eating, food addiction, health professional, clinician

## Abstract

Despite increasing research on the concept of addictive eating, there is currently no published evidence on the views of health professionals who potentially consult with patients presenting with addictive eating behaviours, or of students training to become health professionals. This study aimed to explore the views and understanding of addictive eating behaviours among health professionals and health professionals in training and to identify potential gaps in professional development training. An international online cross-sectional survey was conducted in February–April 2020. The survey (70 questions, 6 key areas) assessed participants’ opinions and clinical experience of addictive eating; opinions on control, responsibility, and stigma relating to addictive eating; and knowledge of addictive eating and opinions on professional development training. In total, 142 health professionals and 33 health professionals in training completed the survey (mean age 38.1 ± 12.5 years, 65% from Australia/16% from the U.K.) Of the health professionals, 47% were dietitians and 16% were psychologists. Most participants (*n* = 126, 72%) reported that they have been asked by individuals about addictive eating. Half of the participants reported that they consider the term food addiction to be stigmatising for individuals (*n* = 88). Sixty percent (*n* = 105) reported that they were interested/very interested in receiving addictive eating training, with the top two preferred formats being online and self-paced, and face-to-face. These results demonstrate that addictive eating is supported by health professionals as they consult with patients presenting with this behaviour, which supports the views of the general community and demonstrates a need for health professional training.

## 1. Introduction

Addictive eating (i.e., an abnormal, recurrent pattern of excessive food consumption despite negative consequences) [1], often referred to as food addiction, is not currently recognised in the Diagnostic and Statistical Manual of Mental Disorders as a distinct diagnosis from other eating and substance use disorders. There exists an ongoing scientific debate in this regard, which centres around whether the symptoms of addictive eating are covered appropriately under other recognised disorders, such as binge eating disorder [2,3]. If addictive eating is a distinct disorder, the debate is also around whether it should be considered a substance (i.e., food) addiction or a behavioural (i.e., eating) addiction, or on a spectrum of overeating [2,3]. Further, there is the question of what the addictive substance/s are or whether it relates to the level of food processing [2,3]. Regardless of whether addictive eating should be recognised, 15–20% of the population report experiencing symptoms that align with addictive eating as determined by self-reported tools [4]. This is higher among certain groups, including females, those with binge eating disorder and other mental health conditions, and those with overweight and obesity [4]. Further, rates of self-perceived addictive eating among community samples range from 27% to 50% [5]. There is also widespread support from community samples that the concept of addictive eating exists [5,6]. For example, a survey of over 600 American and Australian adults reported that 86% believed certain foods may be addictive, and 72% believed addictive eating is linked with an increased risk of obesity [6].

Unhealthy lifestyle behaviours such as poorer dietary intake, physical inactivity, greater time spent sitting, and poor sleep quality are associated with addictive eating [7,8]. This association extends to conditions such as depression, anxiety, and overweight and obesity [9,10]. In terms of clinical management of addictive eating, the published evidence is scarce [11,12]. A recent systematic review conducted by Cassin et al. to assess psychosocial interventions for addictive eating identified only eight studies [12]. Of these, only two studies included individuals with addictive eating and interventions that specifically targeted addictive eating symptoms. The two interventions were abstinence-based (i.e., abstaining from overeating, snacking, and/or from identified problem foods), while the remaining studies included an outcome measure of addictive eating in intervention studies targeting either bulimia nervosa or overweight and obesity. Additionally, all of the included studies were deemed to exhibit poor or fair methodological quality, and most were pilot or feasibility studies. Importantly, the review was limited to psychosocial interventions and did not consider alternate options such as dietary advice alone. Overall, the review’s authors concluded that no effective psychosocial interventions currently exist for the treatment of addictive eating. There is however a high volume of self-help support groups for individuals with addictive eating [13]. A recent review of websites identified 13 online support groups for addictive eating; however only three of these involved credentialed health professionals [13]. Evidently, research exploring the clinical utility of recognising addictive eating as a diagnostic entity and evidence-based best practices for treatment are limited.

There is currently no published evidence on the views of health professionals who likely consult with patients who report addictive eating behaviours, or of those training to become health professionals. Research should examine clinicians’ and future clinicians’ understanding of addictive eating, their support for it as a diagnostic category, and whether professional development training is needed regarding understanding and treating addictive eating. This work is critical to advancing the field of addictive eating in terms of treatment and informing best practice. The aims of this study were to explore the opinions and understanding of addictive eating behaviours among health professionals with experience in weight management and students undertaking relevant health professional training. The study also aimed to explore the needs and preferences for professional development training in addictive eating.

## 2. Materials and Methods

### 2.1. Study Design

An international online cross-sectional survey was conducted. An online survey was used as a convenient method of completion for participants and to maximise the survey reach and response rate. The survey was hosted via Qualtrics (https://www.qualtrics.com/au/) and was open from 21 February to 27 April 2020. The survey took approximately 25 min to complete and was initially pilot tested among a sample of five health professionals and university students to assess for readability and comprehension. The survey consisted of 70 questions including demographic questions and questions across six key areas (opinions and clinical experience of addictive eating; opinions on control, responsibility, and stigma relating to addictive eating; knowledge of addictive eating and opinions on professional development training; opinions on weight gain; treatment of disordered eating and overweight/obesity; and agreement with statements regarding addictive eating symptoms). This paper reports on the questions relating to opinions and clinical experience of addictive eating; opinions on control, responsibility, and stigma relating to addictive eating; and knowledge of addictive eating and opinions on professional development training. Questions relating to the other key areas were outside the scope of the current paper (see File S1 and Table S1). The survey questions used were developed by the research team for the purpose of this study. The survey was set up to require a response to each question before participants could progress to the next question, with the exception of the qualitative questions, which were optional to complete. Survey logic was used so that only relevant questions were displayed to each participant, based on their previous responses. The use of survey logic also limits participants from being able to go back and change previous responses. The study conduct and reporting comply with the Strengthening the Reporting of Observational Studies in Epidemiology (STROBE) guidelines for cross-sectional studies [14]. All participants gave informed consent prior to completing the survey. Participation was voluntary, and no incentives were offered for participation. Ethical approval for the study was obtained from the University of Newcastle Human Research Ethics Committee (H-2019-0349).

### 2.2. Participants and Recruitment

Individuals were eligible to participate if they were a health professional with experience in the management or research of overweight or obesity or disordered eating, or were a student currently enrolled in health professional training at a university. Relevant disciplines included allied health professionals; medical professionals; psychologists; other health professionals; public health, nutrition, or other health researchers; or university students training in one of these professions. University students of relevant disciplines were also included as they represent the next generation of health professionals. Individuals from any country were eligible to participate; however, the survey was written in English. Health professionals and university students completed the same survey; however, some of the survey questions were worded differently by asking health professionals about their practical experience and university students about their opinions. Additionally, the questions regarding experience in treating clients were only asked of health professionals. Recruitment was via convenience sampling and used a range of strategies. Email invitations were sent from the members of the research team to their networks of health professionals and students and contained a link to the online survey. The survey was also advertised via posts from the research team on Twitter, a brief advertisement in the member e-newsletter of Dietitians Australia (professional body for Dietitians in Australia), and an advertisement to students was posted via the online learning management system at the University of Newcastle, Australia. All advertisements used the same recruitment materials and information, which described the survey as a “cross-sectional survey to identify the current understanding of addictive eating behaviours and whether a need exists for professional development training.”

### 2.3. Measures

#### 2.3.1. Demographic Characteristics

Demographic data collected included age, gender, country of residence, and highest qualification completed. Health professionals were also asked their occupation, primary work setting (e.g., hospital, private practice, research), the population group/life stage they primarily work with (e.g., adolescents, adults), and whether they provide advice to individuals with disordered eating or overweight/obesity. University students were also asked the degree for which they were currently studying.

#### 2.3.2. Opinions and Clinical Experience of Addictive Eating

The survey included 14 questions about opinions and clinical experiences regarding addictive eating. Participants were asked whether they had encountered patients/individuals asking or speaking about addictive eating, their thoughts around whether people can develop compulsive eating patterns that resemble an addictive disorder, and whether addictive eating exists (yes, no, or maybe). Of those who indicated that addictive eating does or may exist, participants were asked whether they think different populations may be more vulnerable. Of those who indicated that they provide advice to individuals with disordered eating or overweight/obesity, participants were asked what proportion of their clients may benefit from a specific treatment of addictive eating, if available. Participants were also asked to rate their level of interest in addictive eating becoming a diagnostic term and a referral pathway being introduced for the treatment/management of addictive eating (1/very interested to 5/not at all interested). In terms of treatment for addictive eating, participants were asked their opinion on which health professionals would be best placed to identify and treat people with addictive eating, and which services they would be more/less likely to refer individuals to, as well as whether any particular sub-groups of overweight and obese people would benefit more from a diagnosis of addictive eating. Two open-ended questions were also asked of those who indicated that addictive eating does or may exist, including what they thought were the strengths and weaknesses of using the addictive eating concept to explain eating and weight to individuals.

#### 2.3.3. Opinions on Control, Responsibility, and Stigma Relating to Addictive Eating

Three questions were included relating to opinions on control and responsibility for eating and weight. Participants were asked to rate how much they think it is the responsibility of the individual with addictive eating to gain control over their eating and weight status (1/not responsible to 5/100% responsible) and how much control they think individuals have over their eating and weight (1/a great deal to 5/none at all). Three questions were included relating to their opinions around the different terminology used for addictive eating and stigma. Participants were asked how well they think the term food addiction relates to the experiences of people with weight issues, whether they think the term food addiction is stigmatising, and to indicate which term (if any) they think is most appropriate to describe food addiction/addictive eating.

#### 2.3.4. Knowledge of Addictive Eating and Opinions on Professional Development Training

Three questions asked about participants’ knowledge of addictive eating. Participants were asked what sources of information informed their understanding of addictive eating and to rate their current knowledge of addictive eating and their level of confidence in their knowledge. Six questions asked about participants’ professional development training needs and preferences. Participants were asked about what kinds of professional development training on addictive eating assessment and treatment would be needed, who should receive this training, and their preferred method of training delivery. They were also asked to rate their level of interest in receiving addictive eating training delivered online, whether this would be of interest to colleagues/peers, and whether individuals/clients would be interested in training/management/treatment delivered online.

### 2.4. Analysis

Data were analysed using Stata statistical software version 14.2. In total, 274 individuals accessed the online survey, of these, 175 consented and completed all survey questions (i.e., 64% of those who accessed the survey). Of those that did not complete the survey (*n* = 99), 14 opened the link/viewed the first page but did not start the survey, one did not provide consent and exited the survey, 15 filled in some of the demographics questions only, and the remaining 69 completed the demographics questions and some but not all of the rest of the survey. Quantitative data are reported as number and percentage for categorical variables and mean and standard deviation for continuous variables. Open-response questions are described qualitatively. Qualitative data were analysed using a theoretical thematic analysis approach [15], including (1) identifying codes from the responses based on keywords/phrases, (2) grouping codes into themes, (3) reviewing themes in relation to the contributing codes, and (4) defining and naming themes. One researcher initially conducted the thematic analysis, and this was checked by a second researcher, with any discrepancies discussed and results amended. Themes are presented in the order of most to least frequent/recurrent. Results for health professional and health professional trainee participants were compared using chi-square tests for questions with mutually exclusive response options, to determine whether these were significantly different. There were differences between responses for nine of the questions; however, with further investigation these differences were driven by the large number of response options. As the pattern of the most common responses were similar between the two groups, and due to the small sample size of the health professionals in training, it was deemed appropriate to combine the responses for reporting (see Table S2 for responses by group).

## 3. Results

### 3.1. Sample Characteristics

Of the 175 participants, 81% (*n* = 142) were health professionals, and 19% (*n* = 33) were university students (Table 1). The mean ± SD age of participants was 38.1 ± 12.5 years, the majority were female (*n* = 150, 86%), and participants were from six different countries with most residing in Australia (*n* = 113, 65%) or the U.K. (*n* = 28, 16%). Among the health professional participants, the most common occupations were dietitian (*n* = 66, 47%) and psychologist (*n* = 23, 16%), with the highest proportion working in hospitals (*n* = 39, 28%) and private practice (*n* = 39, 28%), and working with population groups of adults 25–65 years (*n* = 109, 77%) and young adults 18–24 years (*n* = 52, 37%). Sixty-three percent of health professional participants (*n* = 90) reported that they provide advice to clients for disordered eating, while 70% (*n* = 100) provide advice to clients for overweight/obesity.

### 3.2. Description of Quantitative Results

#### 3.2.1. Opinions and Clinical Experience of Addictive Eating

The majority of participants (*n* = 126, 72%) reported that they have been asked by individuals about addictive eating (Table 2). Sixty percent of participants (*n* = 105) indicated that they think addictive eating exists. The proportion of the sample who reported being interested/very interested in addictive eating being a diagnostic term was 48% (*n* = 83).

#### 3.2.2. Opinions on Control, Responsibility, and Stigma Relating to Addictive Eating

The largest proportion of participants reported that they think individuals with addictive eating have “a little” control over their eating habits (*n* = 89, 51%) and weight (*n* = 77, 44%) (Table 3). However, the majority reported that individuals with addictive eating are very/moderately responsible for gaining control over their eating and weight (*n* = 118, 67%). Half of the participants reported that they think food addiction is a stigmatising term for individuals (*n* = 88). Participants’ preferences regarding the terminology used to describe addictive eating/food addiction were varied. From the proposed list of terms, the largest proportion of participants selected compulsive overeating (*n* = 41, 23%), followed by addictive eating (*n* = 34, 19%), and other (*n* = 29, 17%). Of those that selected other, some indicated that eating disorder terminology should be used, some indicated that more than one term is needed as the most appropriate term may differ for different clients/individuals, while other suggested terms included disordered eating, eating addiction, highly processed food addiction, refined food addiction, and restriction–rebound overeating.

#### 3.2.3. Knowledge of Addictive Eating and Opinions on Professional Development Training

The majority of participants rated their knowledge of addictive eating as average or poor (*n* = 106, 61%) (Table 4). The most common source of information that participants used to inform their understanding of addictive eating was colleagues (*n* = 123, 70%), followed by the scientific literature (*n* = 116, 66%). Sixty percent of participants (*n* = 105) reported that they were interested/very interested in receiving training on addictive eating delivered via technologies such as the internet and/or smartphones. When participants were asked who should be trained in addictive eating, the most common responses were dietitians (*n* = 87, 50%) and psychologists (*n* = 82, 47%). In terms of the types of professional development training that is needed, most commonly, participants indicated training in evidence-based treatment (*n* = 142, 81%), followed by understanding medical and non-medical treatments (*n* = 134, 77%) and assessment and diagnosis (*n* = 134, 77%).

#### 3.2.4. Description of Qualitative Results

Thematic analysis results are presented in Table 5. Sixty-three percent (*n* = 111) of the participants responded to the question “What are some strengths/benefits to using the addictive eating approach to explain eating and weight to clients/individuals?” Five themes were identified, including from most to least frequent: (1) provides an explanation/assists understanding; (2) relieves guilt/stigma; (3) provides acknowledgement/validation; (4) provides a framework/pathway for future treatment; and (5) encourages hope for overcoming addictive eating. Fifty-nine percent (*n* = 103) of participants responded to the question “What are some of the downsides/weaknesses to using the addictive eating approach to explain eating and weight to clients/individuals?” Six themes were identified, including from most to least frequent: (1) reason/barrier not to change; (2) negative response from clients/individuals; (3) stigma; (4) lack of evidence/recognition; (5) implications for treatment; and (6) clinician training/time.

## 4. Discussion

This study aimed to explore the opinions and understanding of addictive eating in an international sample of practising health professionals and health professionals in training. The needs and preferences for professional development training in addictive eating were also explored. The majority of the survey sample reported that they support that addictive eating exists, have experienced individuals/patients asking about addictive eating, and expressed interest in receiving training about addictive eating. Overall, the study findings provide important insight into the perspective of currently practicing health professionals and health professionals in training (i.e., future health professionals) on addictive eating. This adds to and provides a point of comparison for the larger evidence base of opinions among the general population.

Sixty percent of the health professionals and health professionals in training surveyed supported that addictive eating exists, while a higher proportion (69%) expressed the view that people can develop compulsive patterns of eating resembling an addictive disorder. These results are substantially lower than in community samples such as the survey by Lee et al. where 86% of adults believed that certain foods may be addictive [6]. Results show that over 70% of health professionals reported that individuals had enquired about addictive eating. Moreover, participants expressed interest in addictive eating being officially recognised as a formal diagnosis and in the use of a specific referral pathway. However, self-rating of knowledge of addictive eating was rated below average in the majority of participants and confidence in knowledge of the evidence base was low. Thus, it is potentially not surprising that our data revealed a definite interest for training and education in this specific topic. Two-thirds of health professionals were interested or very interested in receiving addictive eating training, with almost half reporting that they would prefer training to be online and self-paced and almost half preferring face-to-face training. The most common types of professional development training that were reportedly needed included training in evidence-based treatment, understanding medical and non-medical treatments, and training in assessment and diagnosis. Participants identified dietitians and psychologists as the two major professions who should receive training, followed by psychiatrists and general practitioners, while the majority reported that training in addictive eating would also be useful for individuals or clients. This is not surprising given the pertinent roles that these health professionals have in other recognised forms of disordered eating. These findings indicate that this is a significant issue faced by clients and health professionals.

Overall, there was a mixed response in terms of the preferred terminology to be used to describe this compulsive form of eating. Compulsive overeating was the most preferred term, indicated by 23% of participants, followed by addictive eating (19%). However, a large proportion of participants indicated other responses including that more than one term may be needed as the most appropriate term may differ between clients/individuals. This difference may suggest that a multidimensional or domain-based approach is needed rather than a categorical diagnosis. This also shows that reaching a consensus on a common term may not be achievable. Despite there being a lack of consensus in existing research over the preferred terminology [16,17], the term “food addiction” was the least preferred. This illustrates the recognition amongst those surveyed of the highly stigmatising nature of this descriptor. Indeed, the majority of participants expressed a belief that the term food addiction is stigmatising, which supports consumer research [18]. Many existing research reports discuss the terminology, and it may be time to move beyond the terminology to focus on greater understanding and possible management options, given that many health professionals in the current study have patients seeking help for this behaviour. The qualitative findings from the current study also provide further insight into the discussion of stigma, as this was a recurrent theme when health professionals were asked to explain the benefits and downsides of using the addictive eating approach to explain eating and weight to individuals. Views were divided, in that, some health professionals commented that it may reduce stigma, while others explained that it may introduce the stigma that is associated with addictions and other mental health conditions in general. This may be linked with the number of views expressed about the terminology. Further exploration of the views of health professionals regarding addictive eating and stigma is warranted [19].

The survey identified mixed opinions regarding the relationship between addictive eating behaviours and weight. Over two-thirds of the participants reported that individuals with addictive eating have little to no control over their eating habits and weight. This highlights acceptance of the lack of control experienced by those with addictive eating; yet, approximately half of the participants reported that addictive eating does not relate well to the experiences of people with weight issues. These findings could relate to the fact that individuals may not have been directed to the appropriate services for the management of their addictive eating, i.e., the lack of control relates to numerous unsuccessful attempts at treatment/management by the individual with addictive eating. Comparatively, the study by Lee et al. found that among the community sample of >600 adults, almost three-quarters supported that addictive eating causes obesity, while views were divided close to 50:50 in terms of individuals having control over their weight and eating [6]. These are important findings, as the way that health professionals view these factors would have implications for the treatment that they may provide or refer individuals on to. Further, if these views differ from those of the general population and/or their patients, this may also influence the efficacy of treatment. There has been increasing research of the overlap of disordered eating and obesity [20]. Given that addictive eating often overlaps with binge eating and presents with obesity, this offers an interesting opportunity for further exploration.

The major strength of this study is that it is the first to explore opinions on addictive eating in a sample of health professionals and health professionals in training. Further, a moderate sample size was obtained, which is a strength given the challenges of engaging health professionals in research (e.g., due to busy workloads). The sample was an international sample with health professionals from a range of backgrounds, which is a strength for this exploratory study as it provides a broad range of perspectives; however, the fact that different countries have different professional standards and structures is also a potential limitation. In terms of limitations, health professionals who have an interest in or have been asked about addictive eating may have been motivated to participate in the current study, while a large proportion were dietitians or psychologists. Therefore, the representativeness of the sample is a limitation, and the views presented may not represent the generalised community of health professionals and students. Further, females were over-represented in the study population. However, this can be explained by the higher percentage of women among the health professions surveyed [21] and by the fact that females are more likely to participate in online survey studies than males [22]. The use of convenience sampling is also a limitation in terms of the representativeness of the sample, for example, this likely contributed to the high percentage of dietitians and participants residing in Australia. The survey included a large number and scope of questions as it is the first to explore this topic among health professionals and the intention was to obtain a broad overview of opinions. However, this may have contributed to some participants not completing the survey. Additionally, the survey is based on self-report and, while some qualitative data were collected, the survey included primarily quantitative questions, which may limit the scope of opinions. Many of the participants surveyed also rated their knowledge of addictive eating as below average and their confidence in their knowledge of the latest evidence as low, which could be a limitation to their views on the topic.

The implications of the study findings for research and practice include that practitioners are being asked about addictive eating and there is a need for practitioners to understand addictive eating and the related comorbidities with weight and other mental health conditions such as depression. This would ensure that individuals are provided or directed to the most appropriate service rather than just standard dietary, weight management or psychology advice. One avenue for this could be achieved through professional development training. The focus of professional development training will need to consider the needs of different health professions based on their role in the referral or treatment pathway, for example, focusing on awareness of addictive eating and appropriate services to refer individuals to, compared with evidence-based treatment approaches for those delivering/managing treatment. Despite the lack of consistent terminology, addictive eating may be a means of people seeking help for a mental illness evidenced through having an unhealthy relationship with food. Therefore, there is a need for greater understanding of addictive eating behaviour and possible management options regardless of the terminology that is used to describe it. Future studies should aim to include a varied representation of health professions who may have a role in the care of individuals presenting with addictive eating. For example, GPs who may be the first point of contact for individuals and psychologists, dietitians, or other health professionals who may provide ongoing treatment. As addictive eating is an emerging field of research, health professionals’ views on the topic may change over time, and research into this should be updated accordingly.

## 5. Conclusions

Overall, this survey of an international sample of practising health professionals and health professionals in training identified support for the concept of addictive eating and interest in professional development training. Additional exploration of health professionals’ views on addictive eating is warranted, as this information is critical to advancing the field of addictive eating and informing best practice for assessment and treatment.

## Figures and Tables

**Table 1:** Demographic characteristics of health professionals participating in a survey on addictive eating (*n* = 175).

Demographic Characteristic	*n*	%
**Age (years) Mean ± SD**	38.1 ± 12.5	
Gender		
Female	150	85.7
Male	22	12.6
Other	3	1.7
**Country of residence**		
Australia	113	64.6
U.K.	28	16.0
USA	23	13.1
Other	11	6.3
**Highest qualification completed**		
School certificate/Higher school certificate	21	12.0
Trade or diploma	2	1.1
Undergraduate university degree	50	28.6
Postgraduate university degree	71	40.6
Higher research degree	31	17.7
**Occupation**		
Dietitian	66	37.7
Tertiary health or medical student ^a^	33	18.9
Psychologist	23	13.1
Other health practitioner	18	10.3
Health researcher	12	6.9
Tertiary academic/teacher	6	3.4
Medical specialist/registrar	4	1.7
General practitioner	3	1.7
Counsellor	3	1.7
Pharmacist	3	1.7
Psychotherapist	2	1.1
Social worker	2	1.1
**Primary work situation ^b^**		
Hospital	39	27.5
Private practice	39	27.5
Research and teaching	29	20.4
Community/population/public health program	19	13.4
Primary care	7	4.9
Food service	1	0.7
Other	8	5.6
**Population group worked with ^b,c^**		
Infants < 2 years	13	9.2
Children 2–12 years	20	14.1
Adolescents 13–17 years	39	23.2
Young adults 18–24 years	52	36.6
Adults 25–65 years	109	76.8
Adults > 65 years	41	28.9
Not applicable	4	2.8

^a^ Of the tertiary health and medical students, *n* = 29 (88%) were studying a degree in Nutrition and Dietetics. ^b^ Responses are for health professionals only (*n* = 142). ^c^ Multiple response question i.e., percentages add to >100.

**Table 2:** Opinions and clinical experience of addictive eating among health professionals participating in a survey on addictive eating (*n* = 175).

Survey Item	*n*	%
**Have you experienced individuals asking or speaking about addictive eating?**		
Yes	126	72.0
Maybe	14	8.0
No	35	20.0
**In your opinion, do you feel that people can develop compulsive patterns of eating that resemble an addictive disorder?**		
Yes	120	68.6
Maybe	33	18.9
No	22	12.6
**In your opinion, does addictive eating exist?**		
Yes	105	60.0
Maybe	33	18.9
No	37	21.1
**In your opinion, do you feel that there are population groups who may be more vulnerable to addictive eating? ^a^**		
Yes	75	54.3
Unsure	18	13.0
No	45	32.6
**Estimated percentage of clients to benefit from a specific treatment of addictive eating (Mean SD) ^b^**	40.9 ± 27.9	
**How interested would you be in addictive eating being a diagnostic term?**		
Very interested	43	24.6
Interested	40	22.9
Somewhat interested	29	16.6
Not very interested	23	13.1
Not at all interested	40	22.9
**How interested would you be if there was a referral pathway for the treatment/management of addictive eating?**		
Very interested	72	41.1
Interested	41	23.4
Somewhat interested	20	11.4
Not very interested	6	3.4
Not at all interested	36	20.6
**Who do you think would be best placed to identify people with behaviours suggestive of addictive eating? ^c^**		
Dietitians/nutritionists	99	56.6
Psychologists	93	53.1
Psychiatrists	51	29.1
Counsellor	49	28.0
General practitioner	48	27.4
Medical specialists	30	17.1
All of the above	75	42.9
Other	30	17.1
**Who do you think is best placed to provide treatment for people with addictive eating?** ^c^		
Psychologists	114	65.1
Dietitians/nutritionists	107	61.1
Psychiatrists	52	29.7
Counsellor	49	28.0
General practitioner	16	9.1
Medical specialists	17	9.7
All of the above	34	19.4
Other	29	16.6
**Are there any services you would be more likely to refer to or suggest to clients/individuals with addictive eating? ^c^**		
Psychologist	124	70.9
Counselling	77	44.0
Addiction specialist	75	42.9
General practitioner	19	10.9
Pharmacological	8	4.6
All of the above	96	54.9
Other	14	8.0
None	34	19.4
**Are there any services you would be less likely to refer to or suggest to clients/individuals with addictive eating? ^c^**		
Pharmacological	86	49.1
General practitioner	76	43.4
Addiction specialist	33	18.9
Counselling	6	3.4
Psychologist	2	1.1
All of the above	7	4.0
Other	3	1.7
None	46	26.3
**Are there any particular sub-groups of overweight and obese people you feel would benefit more from a diagnosis of addictive eating? ^c^**		
Individuals with binge eating disorder	80	45.7
Overeaters	79	45.1
Individuals with a mental health condition	60	34.3
Individuals with other mental illnesses	44	25.1
Individuals with substance disorders	36	20.6
Individuals with low motivation to engage with treatment	30	17.1
Children	14	8.0
Other	17	9.7
No	58	33.1

^a^*n* = 138 responses (i.e., those that believe addictive eating exists). ^b^ *n* = 80 responses from health professionals (i.e., those that believe addictive eating exists and provide treatment for overweight/obesity and/or disordered eating). ^c^ Multiple response questions, i.e., percentages add to >100.

**Table 3:** Opinions on control, responsibility, and stigma relating to addictive eating among health professionals participating in a survey on addictive eating (*n* = 175).

Survey Item	*n*	%
**In your opinion, how much control does someone with addictive eating have over their eating habits?**		
A great deal	5	2.9
A lot	9	5.1
A moderate amount	60	34.3
A little	89	50.9
None at all	12	6.9
**In your opinion, how much control does someone with addictive eating have over their weight?**		
A great deal	2	1.1
A lot	2	1.1
A moderate amount	44	25.1
A little	77	44.0
None at all	50	28.6
**In your opinion, how much responsibility does someone with addictive eating have to gain control over their eating and weight?**		
100% responsible	12	6.9
Very responsible	51	29.1
Moderately responsible	67	38.3
Not very responsible	20	11.4
Not responsible	25	14.3
**Do you think that the term “food addiction” is stigmatising for individuals?**		
Yes	88	50.3
Unsure	52	29.7
No	35	20.0
**How well do you think the term food addiction relates to the experiences of people with weight issues?**		
Extremely/very well	59	33.7
Neutral	35	20.0
Not well	81	46.3
**Select which term you feel is most appropriate to describe food addiction/addictive eating?**		
Compulsive overeating	41	23.4
Addictive eating	34	19.4
Compulsive overeating disorder	27	15.4
Food addiction	23	13.1
None, no term needed	21	12.0
Other	29	16.6

**Table 4:** Knowledge of addictive eating and opinions on professional development training among health professionals participating in a survey on addictive eating (*n* = 175).

Survey Item	*n*	%
**How confident do you feel in your knowledge on the latest evidence relating to addictive eating (i.e., assessment methodologies/treatment)?**		
Extremely confident	26	14.9
Very confident	26	14.9
Neutral	41	23.4
Somewhat confident	34	19.4
Not at all confident	48	27.4
**How would you rate your current knowledge about addictive eating?**		
Excellent	30	17.1
Good	36	20.6
Average	57	32.6
Poor	49	28.0
Terrible	3	1.7
**What sources of information have informed your understanding of addictive eating? ^a^**		
Colleagues	123	70.3
Scientific literature	116	66.3
Education	102	58.3
Conferences	68	38.9
Social media	36	20.6
Other reading	27	15.4
Traditional media	21	12.0
Have not heard of addictive eating	7	4.0
**If training for addictive eating were available, how interested would you be in participating in training delivered using technologies such as the internet and/or smartphones?**		
Very interested	75	42.9
Interested	30	17.1
Somewhat interested	24	13.7
Not very interested	10	5.7
Not at all interested	36	20.6
**In your opinion, who should be trained in addictive eating assessment and treatment? ^a^**		
Dietitians	87	49.7
Psychologists	82	46.9
Psychiatrists	55	31.4
General practitioners	52	29.7
Undergraduate students	38	21.7
Medical specialists	33	18.9
Practice nurses	25	14.3
All of the above	73	41.7
Other	38	21.7
**If food addiction/addictive eating became a diagnostic term, what kinds of professional development training do you think would be needed (for yourself/other professions)? ^a^**		
Evidence-based treatment	142	81.1
Understanding treatment (medical and non-medical)	134	76.6
Assessment/diagnosis	134	76.6
Treatment approaches focusing on other behaviours as well as food, e.g., sleep, physical activity	129	73.7
Understanding addiction terminology	123	70.3
Neuroscience behind addictive eating	119	68.0
How to minimise stigma	114	65.1
Foods to avoid	59	33.7
Other	36	20.6
**What would be your preferred method of delivery for professional development training? ^b^**		
Face to face	81	46.3
Online, self-paced	77	44.0
Professional development	65	37.1
Structured short course	63	36.0
Delivered by a credential source	51	29.1
Other	13	7.4
**Do you think online training/management/treatment delivered by health professionals would be of interest to clients/individuals?**		
Yes/Maybe	157	89.7
No	18	10.3
**Do you think online training would be of interest to your co-workers/colleagues/peers?**		
Yes/Maybe	154	88.0
No	21	12.0

^a^ Multiple response questions i.e., percentages add to >100. ^b^ Reported as the *n*(%) who ranked responses as 1 or 2.

**Table 5:** Qualitative findings among health professionals participating in a survey on addictive eating (*n* = 175).

**Question: What are some strengths/benefits to using the addictive eating approach to explain eating and weight to clients/individuals?**	
Themes and quotes	(1) Provides an explanation/assists understanding “Help clients realise the link between behaviours, thoughts, and food…” “Help people understand the role of psychology in food choice”
	(2) Relieves guilt/stigma “May help to reduce stigma and some of the extreme negative thoughts people have in relation to their eating.” “Clients may feel less guilty about weight/weight gain.”
	(3) Provides acknowledgement/validation “Legitimises their problem” “‘Giving it a name’ may help people externalise and tackle the issue better.”
	(4) Provides a framework/pathway for future treatment “Current knowledge about addiction medicine would provide potential avenues for treatment.”
	(5) Encourages hope for overcoming addictive eating “When they [clients] feel understanding and empowered it is easier to facilitate health promoting changes and more effective strategies.”
**Question: What are some of the downsides/weaknesses to using the addictive eating approach to explain eating and weight to clients/individuals?**	
Themes and quotes	(1) Reason/barrier not to change “Some people may like another label as a reason not to try to change.” “Dissolves some responsibility for lifestyle decisions that are outside of addictive behaviours.”
	(2) Negative response from clients/individuals “[It] may induce a sense of helplessness.” “Some people may get offended when using the word addictive, may bring up deep rooted emotional issues associated with why they overeat.”
	(3) Stigma “It can become a stigmatised label of being an ‘addict’, which may impact on their recovery journey.”
	(4) Lack of evidence/recognition “I do not see this [food addiction] at the moment as true addiction.” “The fact that scientific literature and other health care professionals don’t support this.”
	(5) Implications for treatment “The abstinence model may have the potential to increase binge eating if it is too restrictive regarding food rules.” “Limited psychological support to help manage the condition”
	(6) Clinician training/time “Clinicians need to be trained to identify and safely address addictive eating … Identifying the eating behaviour without appropriate treatment may be detrimental.”

Questions were only asked of those participants who responded yes or maybe to the question, “do you believe addictive eating exists?”

## Supplementary Materials

The following are available online at https://www.mdpi.com/2072-6643/12/9/2860/s1, File S1: Health professionals’ and health professional trainees’ views on addictive eating behaviours: A cross sectional survey - Additional results, Table S1: Opinions on weight gain, and treatment of individuals with disordered eating or overweight/obesity among health professionals participating in a survey on addictive eating (*n* = 175), Table S2: Responses of participants in a survey on addictive eating, by health professionals and health professionals in training (*n* = 175).
