# Supplementary material for: Health Professionals’ and Health Professional Trainees’ Views on Addictive Eating Behaviours: A Cross-Sectional Survey
Source: Nutrients. 2020 Sep 18;12(9):2860. doi: 10.3390/nu12092860 (PMC7551788; doi:10.3390/nu12092860)
Supplement: Supplementary file 1 [file nutrients-12-02860-s001.pdf]

Supplementary File:

Health professionals' and health professional trainees' views on addictive eating behaviours: A cross sectional survey - Additional results

Methods

Opinions on weight gain

Participants were asked to indicate the factors they think contribute to an individual gaining weight from a list of 8 predetermined options *(e.g. cultural, psychological, lifestyle, parental influences, public policies).*

Treatment of individuals with disordered eating or overweight/obesity

Participants were asked whether they provide advice to individuals with disordered eating or overweight/obesity, and whether they refer individuals with disordered eating or overweight/obesity to other health practitioners *(yes, no or not applicable).* Those that had provided advice were asked to indicate the types of assistance they provide to individuals with binge eating disorder and overweight/obesity separately from a list of 12 predetermined options or 'other, please specify'. They were also asked to rank the methods they believe are most effective for treatment/management from a selection of 6 methods or 'other, please specify'. Participants who had referred individuals to other health practitioners were asked to indicate the types of treatments they provide or refer to for individuals with binge eating disorder and overweight/obesity from a list of 6 predetermined options or 'other, please specify'. Two questions asked participants to rank potential barriers to delivering optimal treatment for weight management and for binge eating disorder from a selection of 6 barriers or 'other, please specify'.

Results

The major factors participants' reported that may contribute to weight gain were psychological factors and genetic/biomedical factors (n=131, 75% each), followed by environmental factors (n=130, 74%) (Supplementary Table 1). Among health professionals who provide treatment to clients, the most common type of treatment provided to clients with either overweight/obesity or binge eating disorder was behaviour change advice, reported by 77% (n=77) and 76% (n=68) of participants respectively.

Supplementary Table 1 Opinions on weight gain, and treatment of individuals with disordered eating or overweight/obesity among health professionals participating in a survey on addictive eating (n=175)

	N	%		N	%
Opinions on weight gain					
**Factors that may contribute to weight gain**					
Psychological factors	131	74.9			
Genetic/biomedical factors	131	74.9			
Environmental factors	130	74.3			
Lifestyle factors	128	73.1			
Parental influences/childhood	126	72.0			
Social factors	117	66.9			
Cultural factors	103	58.9			
Public policies	69	39.4			
Experience/Opinions in treating disordered eating and overweight/obesity					
**Types of treatment provided to clients with overweight/obesity** [a, b]			**Types of treatment provided to clients with binge eating disorder** [a, e]		
Behaviour change advice/strategies	77	77.0		68	75.6
Healthy eating education	76	76.0		52	57.8
Self-monitoring intake	51	51.0		51	56.7
Refer to another health professional	40	40.0		36	40.0
Psychological counselling	31	31.0		32	35.6
Very low energy meal replacements	26	26.0		16	17.8
Meal plan for 0.5-1kg/week weight loss	25	25.0		6	6.7
Bariatric surgery	24	24.0		5	5.6
Exercise regime	22	22.0		3	3.3
Medications	11	11.0		2	2.2
Combination of treatments	11	11.0		2	2.2
Commercial weight loss programs	8	8.0		1	1.1
Other	20	20.0		20	22.2
**Most effective methods for weight management** [b, c, d]			**Most effective methods for treating binge eating disorder** [c, d, e]		
Diet	72	56.7		23	19.7

Combination of treatments	67	52.8	66	56.4
Therapy/counselling	32	25.2	104	88.9
Exercise	28	22.0	1	0.9
Surgery	27	21.3	3	2.6
Prescription drugs	3	2.4	7	6.0
Other	25	19.7	30	25.6
**Barriers to delivering weight management treatment**			**Barriers to delivering binge eating disorder treatment**	
Time restrictions for clinicians	68	38.9	64	36.6
Time restrictions for clients	66	37.7	56	32.0
Waiting lists	62	35.4	84	48.0
Limited effective options	67	38.3	57	32.6
No access to services	49	28.0	35	20.0
Cost to clients	82	46.9	77	44.0
Other	61	34.9	56	32.0
**Types of treatment provided/referred to for clients with overweight/obesity for weight management** [a, e]			**Types of treatment provided/referred to for clients with binge eating disorder** [a, f]	
Diet	54	77.1	43	46.7
Exercise	46	65.7	23	25.0
Prescription drugs	16	22.9	10	10.9
Surgery	17	24.3	3	3.3
Therapy/counselling	49	70.0	73	79.3
Combination of treatments	15	21.4	14	15.2
Other	7	10.0	17	18.5

Responses where percentages add to >100 were multiple response questions. [a] Responses are for health professionals only. [b] N=100 responses from health professionals (i.e. those who indicated they provide treatment for overweight/obesity). [c] reported as the N(%) who ranked responses as 1 or 2. [d] N=27 responses from health students. [e] N=90 responses from health professionals (i.e. those who indicated they provide treatment for binge eating disorder). [f] N=70 responses from health professionals.

Supplementary Table 2. Responses of participants in a survey on addictive eating, by health professionals and health professionals in training (n=175)

	Health professionals (n=142)		Health professionals in training (n=33)	
	N	%	N	%
**Have you experienced individuals asking or speaking about addictive eating?**				
Yes	102	71.8	24	72.7
Maybe	11	7.7	3	9.1
No	29	20.4	6	18.2
**In your opinion, do you feel that people can develop compulsive patterns of eating that resemble an addictive disorder?** [a]				
Yes	91	64.1	29	87.9
Maybe	30	21.1	3	9.1
No	21	14.8	1	3.0
**In your opinion, does addictive eating exist?** [a]				
Yes	76	53.5	29	87.9
Maybe	31	21.8	2	6.1
No	35	24.6	2	6.1
**In your opinion, do you feel that there are population group/s who may be more vulnerable to addictive eating?** [a, b]				
Yes	56	52.3	19	61.3
Unsure	6	5.6	12	38.7
No	45	42.1	0	0.0
**How interested would you be in addictive eating being a diagnostic term?** [a]				
Very interested	30	21.1	13	39.4
Interested	30	21.1	10	30.3
Somewhat interested	24	16.9	5	15.2
Not very interested	20	14.1	3	9.1
Not at all interested	38	26.8	2	6.1
**How interested would you be if there was a referral pathway for the treatment/management of addictive eating?** [a]				
Very interested	55	38.7	17	51.5
Interested	30	21.1	11	33.3
Somewhat interested	18	12.7	2	6.1
Not very interested	4	2.8	2	6.1
Not at all interested	35	24.7	1	3.0

**Who do you think would be best placed to identify people with behaviours suggestive of addictive eating?** [c]				
General Practitioner	38	26.8	10	30.3
Medical specialists	20	14.1	10	30.3
Psychologists	69	48.6	24	72.7
Psychiatrists	36	25.4	15	45.5
Counsellor	33	23.2	16	48.5
Dietitians/Nutritionists	74	52.1	25	75.8
All of the above	59	41.6	16	48.5
Other	28	19.7	2	6.1
**Who do you think is best placed to provide treatment for people with addictive eating?** [c]				
General Practitioner	11	7.8	5	15.2
Medical specialists	12	8.5	5	15.2
Psychologists	91	64.1	23	69.7
Psychiatrists	39	27.5	13	39.4
Counsellor	43	30.3	6	18.2
Dietitians/Nutritionists	84	59.2	23	69.7
All of the above	28	19.7	6	18.2
Other	27	19.0	2	6.1
**Are there any services you would be more likely to refer to or suggest to clients/individuals with addictive eating?** [c]				
Counselling	62	43.7	15	45.5
Psychologist	98	69.0	26	78.8
Addiction specialist	54	38.0	21	63.6
Pharmacological	7	4.9	1	3.0
General Practitioner	17	12.0	2	6.1
All of the above	72	50.7	24	72.7
Other	11	7.8	3	9.1
None	30	21.1	4	12.1
**Are there any services you would be less likely to refer to or suggest to clients/individuals with addictive eating?** [c]				
Counselling	6	4.2	0	0.0
Psychologist	2	1.4	0	0.0
Addiction specialist	33	23.2	0	0.0
Pharmacological	65	45.8	21	63.6
General Practitioner	58	40.9	18	54.6

All of the above	7	4.9	0	0.0
Other	2	1.4	1	3.0
None	46	32.4	0	0.0
**Are there any particular sub-groups of overweight and obese people you feel would benefit more from a diagnosis of addictive eating?** [c]				
Overeaters	60	42.3	19	57.6
Individuals with binge eating disorder	59	41.6	21	63.6
Children	7	4.9	7	21.2
Individuals with low motivation to engage with treatment	23	16.2	7	21.2
Individuals with a mental health condition	43	30.3	17	51.5
Individuals with other mental illnesses	30	21.1	14	42.4
Individuals with substance disorders	29	20.4	7	21.2
Other	16	11.3	1	3.0
No	54	38.0	4	12.1
**In your opinion, how much control does someone with addictive eating have over their eating habits?**				
A great deal	5	3.5	0	0.0
A lot	7	4.9	2	6.1
A moderate amount	50	35.2	10	30.3
A little	70	49.3	19	57.6
None at all	10	7.0	2	6.1
**In your opinion, how much control does someone with addictive eating have over their weight?**				
A great deal	2	1.4	0	0.0
A lot	1	0.7	1	3.0
A moderate amount	32	22.5	12	36.4
A little	62	43.7	15	45.5
None at all	45	31.7	5	15.2
**In your opinion, how much responsibility does someone with addictive eating have to gain control over their eating and weight?** [a]				
100% responsible	6	4.2	6	18.2
Very responsible	38	26.8	13	39.4
Moderately responsible	56	39.4	11	33.3
Not very responsible	18	12.7	2	6.1
Not responsible	24	16.9	1	3.0
**Do you think that the term 'food addiction' is stigmatizing for individuals?**				
Yes	76	53.5	12	36.4

Unsure	41	28.9	11	33.3
No	25	17.6	10	30.3
**How well do you think the term food addiction relates to the experiences of people with weight issues?** [a]				
Extremely well	10	7.0	3	9.1
Very well	31	21.8	15	45.5
Neutral	28	19.7	7	21.2
Somewhat well	33	23.2	6	18.2
Not at all well	40	28.2	2	6.1
**Select which term you feel is most appropriate to describe food addiction/addictive eating?**				
Food addiction	17	12.0	6	18.2
Addictive eating	25	17.6	9	27.3
Compulsive overeating	33	23.2	8	24.2
Compulsive overeating disorder	20	14.1	7	21.2
Other	27	19.0	2	6.1
None, no term needed	20	14.1	1	3.0
**How confident do you feel in your knowledge on the latest evidence relating to addictive eating (i.e. assessment methodologies/treatment)?** [a]				
Extremely confident	26	18.3	0	0.0
Very confident	24	16.9	2	6.1
Neutral	30	21.1	11	33.3
Somewhat confident	25	17.6	9	27.3
Not at all confident	37	26.1	11	33.3
**How would you rate your current knowledge about addictive eating?** [a]				
Excellent	29	20.4	1	3.0
Good	31	21.8	5	15.2
Average	49	34.5	8	24.2
Poor	30	21.1	19	57.6
Terrible	3	2.1	0	0.0
**What sources of information have informed your understanding of addictive eating?** [c]				
Education	83	58.5	19	57.6
Traditional media	13	9.2	8	24.2
Social media	18	12.7	18	54.6
Scientific literature	100	70.4	16	48.5
Conferences	63	44.4	5	15.2

Colleagues	108	76.1	15	45.5
Other reading	24	16.9	3	9.1
Have not heard of addictive eating	4	2.8	3	9.1
**If training for addictive eating was available, how interested would you be in participating in training delivered using technologies such as the internet and/or smartphones?**				
Very interested	59	41.6	16	48.5
Interested	23	16.2	7	21.2
Somewhat interested	17	12.0	7	21.2
Not very interested	8	5.6	2	6.1
Not at all interested	35	24.7	1	3.0
**In your opinion, who should be trained in addictive eating assessment and treatment?** [c]				
Undergraduate students	21	14.8	17	48.5
Practice nurses	18	12.7	7	21.2
Dietitians	65	45.8	22	66.7
Psychologists	61	43.0	21	63.6
Psychiatrists	41	28.9	14	42.4
General Practitioners	42	29.6	10	30.3
Medical Specialists	24	16.9	9	27.3
All of the above	57	40.1	16	48.5
Other	37	26.1	1	3.0
**If food addiction/addictive eating became a diagnostic term, what kinds of professional development training do you think would be needed (for yourself/other professions)?** [c]				
Understanding addiction terminology	93	65.5	30	90.9
Understanding treatment (medical & non-medical)	104	73.2	30	90.9
Assessment/diagnosis	104	73.2	30	90.9
Evidence-based treatment	112	78.9	30	90.9
How to minimise stigma	94	66.2	20	60.6
Neuroscience behind addictive eating	91	64.1	28	84.9
Foods to avoid	49	34.5	10	30.3
Behavioural approaches as well as food e.g. sleep	98	69.0	31	93.9
Other	34	23.9	2	6.1
**What would be your preferred method of delivery of professional development training?** [c, d]				
Online, self-paced	69	48.6	8	24.2

Face to face	66	46.5	15	45.5
Structured short course	44	31.0	19	57.6
Professional development	55	38.7	10	30.3
Delivered by a credential source	37	26.1	14	42.4
Other	13	9.2	0	0.0
**Do you think online training/management/treatment delivered by health professionals would be of interest to clients/individuals?**				
Yes	78	54.9	23	69.7
Maybe	48	33.8	8	24.2
No	16	11.3	2	6.1
**Do you think online training would be of interest to your co-workers/colleagues/peers?**				
Yes	78	54.9	22	66.7
Maybe	46	32.4	8	24.2
No	18	12.7	3	9.1

[a] Indicates statistically significant difference in responses between health professionals and health professionals in training assessed by chi square test ($p<0.05$). [b] N=138 responses (i.e. those that believe addictive eating exists). [c] Multiple response questions i.e. percentages add to >100. Chi square tests to determine significant differences were not performed for these questions as the responses are not mutually exclusive (i.e. test assumptions not met). [d] Reported as the N(%) who ranked responses as 1 or 2.
